# Pediatric Central Nervous System Tumors: State-of-the-Art and Debated Aspects

**DOI:** 10.3389/fped.2018.00309

**Published:** 2018-11-01

**Authors:** Mitchell T. Foster, Lalgudi Srinivasan Harishchandra, Conor Mallucci

**Affiliations:** Department of Neurosurgery, Alder Hey NHS Foundation Trust, Liverpool, United Kingdom

**Keywords:** intraoperative magnetic resonance imaging, pediatric neuroimaging, technology in surgery, neurooncology, pediatric brain tumors

## Abstract

Pediatric neuro-oncology surgery continues to progress in sophistication, largely driven by advances in technology used to aid the following aspects of surgery: operative planning (advanced MRI techniques including fMRI and DTI), intraoperative navigation [preoperative MRI, intra-operative MRI (ioMRI) and intra-operative ultrasound (ioUS)], tumor visualization (microscopy, endoscopy, fluorescence), tumor resection techniques (ultrasonic aspirator, micro-instruments, micro-endoscopic instruments), delineation of the resection extent (ioMRI, ioUS, and fluorescence), and intraoperative safety (neurophysiological monitoring, ioMRI). This article discusses the aforementioned technological advances, and their multimodal use to optimize safe pediatric neuro-oncology surgery.

## Introduction

Following clinical and radiological diagnosis, the surgical management of pediatric brain tumors involves tumor biopsy, tumor excision, and the management of perioperative surgical complications including CSF diversion. There is a balance between maximizing surgical resection whilst minimizing surgical morbidity. This has to be balanced with post-operative plans for further oncological treatment, and the natural history of the tumor in question. Advances in preoperative and operative tools and techniques help to optimize this process of perioperative decision making and the operative intervention itself. A full discussion of pre-operative workup is beyond the scope of this article. In short, MRI techniques & radiologist expertise have evolved such that preoperative prediction of not only the tumor histology but also molecular subgroup is increasingly accurate (1, 2).

## Operative intervention

### Tumor biopsy

Biopsy may be performed with an open technique (through craniotomy), through a small burr hole, or endoscopically.

The precision of biopsy has evolved over time with progression of technology: first open biopsy, then stereotactic frame biopsy, then frameless biopsy which continues to evolve in sophistication, including the use of robotic assisted biopsy which is now becoming mainstream (3).

Neuro-navigation is used to plan biopsy entry sites, trajectory and the intracranial target. This can be done with precision to avoid eloquent gray or white matter and vessels within the trajectory.

Volume MRI is used alongside stereotactic frame, optical neuro-navigation, or electromagnetic neuro-navigation (4). Stereotactic frame and optical techniques require pin fixation to the skull; this makes them more accurate, but contraindicated in infants. Electromagnetic navigation allows head movement, is not dependant on skull pin fixation, but as a consequence has less precision. Optical and electromagnetic techniques allow real-time tracking of the biopsy tip during the procedure (5).

In cases where the surgical target is small, intraoperative MRI can be used to confirm the biopsy site's accuracy to the planned target prior to waking the patient, should further biopsy be required.

### Tumor excision

#### Planning

The surgical plan may be for tumor debulking or complete excision. Within this range, there is complex decision making to maximize safe resection whilst minimizing morbidity which depends on two main factors: tumor type (influencing propensity for recurrence, metastasis, overall survival and the need for adjuvant therapies), and tumor location (proximity to eloquent areas of brain and vital neurovascular, or neuroendocrine structures).

**Figure 1:**
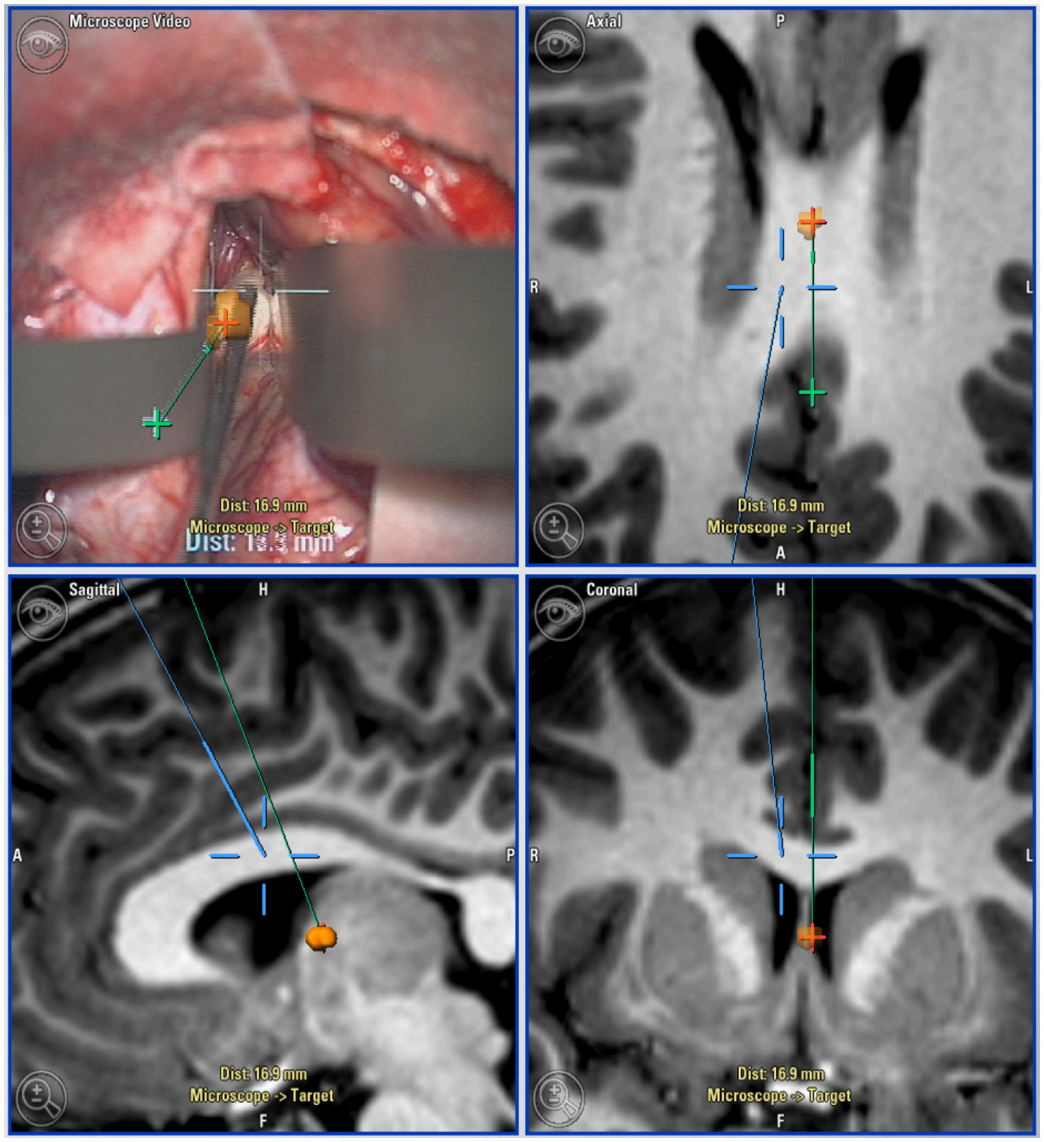
Neuro-navigation guided surgical approach: Top left: microscope view with navigation overlaid. Top right (axial), Bottom left (sagittal), and bottom right (coronal): Views of target lesion with planned trajectory (green) and microscope line of sight (blue).

Software can be used to mark out the tumor extent and a resection plan which can be used intraoperatively to navigate to the tumor. Functional MRI can be used to identify eloquent areas involved in motor generation, speech and language (6). The applicability of fMRI in pediatric neurosurgery is limited because it needs patient cooperation and some form of sedation, especially in children under 6 years (7). Diffusion weighted MRI can be processed using diffusion tensor imaging based tractography to map out white matter tracts in the vicinity of the resection cavity (8). Tracts may be for example within tumor, abutting the tumor, or split by the tumor, which may impact the decision of both resection extent and direction of approach (Figure 1). DTI has the disadvantage that it does not offer any functional information, and based on the *post-hoc* analysis it can be represented in several ways (9).

In complex intrinsic tumors, multi-voxel MR spectroscopy is used to identify the most aggressive components and aid resection planning (Figure 1).

Preoperative volume MRI, once registered for neuro-navigation can be used to plan an optimal skin incision and craniotomy on the patient on the table (10).

#### Operative microscope

The history and vital role of the microscope in neuro-oncology surgery is well described elsewhere (11). It provides magnification and illumination, while allowing ergonomic movement to resect tumors with increased precision. Each successive iteration of microscope has improved their functionality and utility. The most recent advances include 3D stereoscopic visualization to an external screen for the operating surgeon if desired, assistant(s) and theater staff (12). This is beneficial for safety, as the non-operating surgical team can visualize the current surgical activity in real-time, and respond promptly to problems such as hemorrhage. This also benefits teaching within the department. Another development is angled micro-endoscopy (12). This system permits visualization around corners, and has been used with success in brainstem tumor resection in our department.

#### Navigation

Neuro-navigation can be used for tumor biopsy as discussed above, and for operative planning for resection as described above. Intraoperatively, it is used to confirm the location of tumor alongside normal anatomy. Plans made preoperatively which include anatomical regions of interest, fMRI, or tractography can be used in real-time to ensure maximal safety.

**Figure 2:**
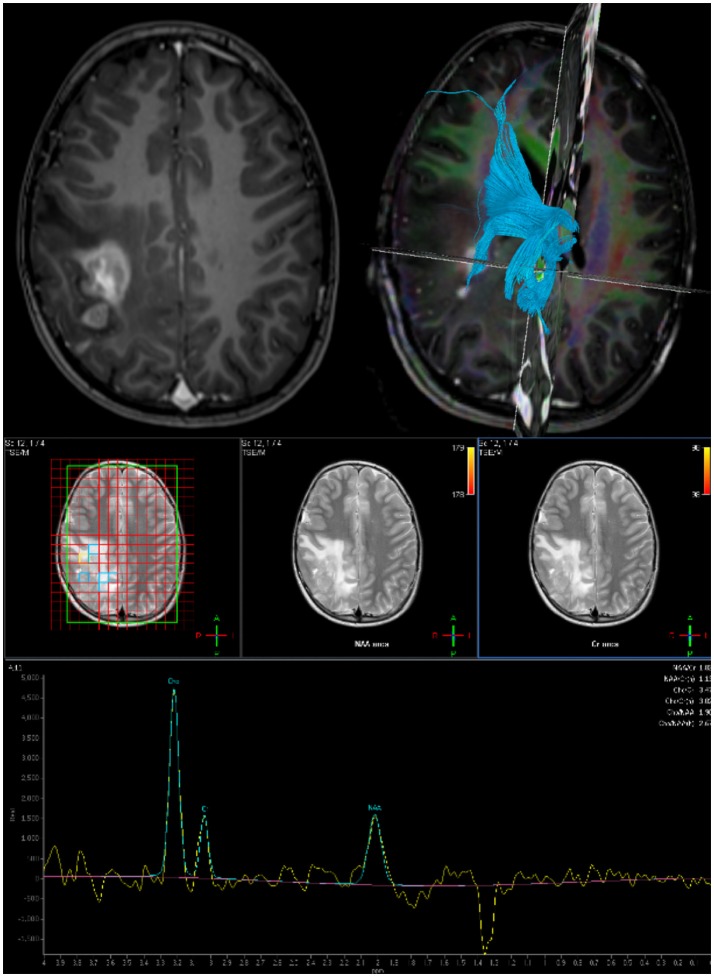
Preoperative imaging for planning surgical approach and resection extent: **Top Left:** Axial post gadolinium T1 MRI. **Top Right:** DTI derived tractography to map white matter bundles in vicinity of tumor. **Bottom:** Multi-voxel MR Spectroscopy to define resection target.

A navigated “pointer” is the most commonly used tool. In addition, the operative microscope can be integrated with the neuro-navigation system such that the point of maximal focus (indicated by the convergence of two laser pointers in the operative field) becomes visible on the navigation screens. It is also possible to overlay navigation onto the microscope view to the surgeon (Figure 2). Recently, a navigable suction catheter has been developed to allow synchronous tumor resection with navigation, reducing the need to continually re-site a navigation pointer.

Navigation has become a standard technique for pediatric neuro-oncological surgery, and indeed much of all cranial neurosurgery. However it is not without limitations: It is subject to error during the fusion of different image sequences, and at the point of registration to the patient. Once craniotomy and durotomy have been performed, the brain will be permitted to shift. The variable degree to which this occurs can unpredictably diminish navigation's accuracy to the intracranial anatomy. This inaccuracy increases with progressive tumor resection as the brain shifts more. Operating theaters can be crowded with staff and equipment; it can be challenging to ensure the navigation camera's field of view can see the optical reference points at all times.

Furthermore, two recent Cochrane reviews have highlighted the lack of evidence for intraoperative neuro-navigation in terms of tumor resection and quality of life (13, 14). While there is a lack of evidence in support of these techniques, there is no evidence against, and most surgeons would agree, this is an invaluable resource that is a mainstay of neuro-oncology surgery (11).

There is anticipated to be a greater role for “augmented reality” techniques in neuro-navigation as technology progresses (15).

#### Intraoperative MRI

Tejada et al provide a comprehensive summary of intraoperative MRI at our center, with the largest published series of ioMRI tumor resections (16).

The patient's head is placed in a non-magnetic frame with (or without) pin fixation. The patient is registered to their preoperative volume MRI and the operation is performed. After tumor resection, at a point of safety with adequate hemostasis, the skin is loosely approximated, and the wound is draped to ensure sterility. The MRI coil head is placed over the drapes and secured. The patient is then transferred to the neighboring ioMRI room, scanned, and returned to the operative theater. Optical markers on the MRI coil allows the patient to be automatically re-registered to the new intraoperative scan.

The choice of MR sequence is determined by the pre-operative findings. For example, a low grade tumor that does not enhance on T1 MRI with gadolinium, but is visible on FLAIR sequences, would have this sequence performed intraoperatively, and then mapped onto an intraoperative volume T1 sequence for intraoperative navigation. Images are reviewed with a consultant neuro-radiologist familiar with the case and intraoperative MRI alongside the operating surgeon.

IoMRI may occasionally be performed after the wound has been closed: this occurs in cases when the surgeon is confident of the surgical resection, or if further resection is thought to be too high risk for post-operative morbidity. This is logistically advantageous for theater efficiency, but still preserves sterility of the field, in case the MRI identifies anything requiring further surgery (for example unexpected resectable residual tumor or haematoma). If as predicted, no further surgery is required, the patient can then simply be undraped and woken. This technique can also be done for biopsy cases, to confirm the target site. In most cases, this will serve as the immediate post-operative scan, and the patient will proceed to extubation and recovery. However, the patient remains within the ioMRI frame, to permit automatic re-registration to the ioMRI, for re-opening of the wound if deemed necessary (for example for further tumor resection, repeat biopsy or hemorrhage control).

There are multiple advantages to ioMRI: It allows diagnosis of residual tumor and surgical damage (ischemia, or hemorrhage). It allows automatic reregistration to an accurate scan to correct for brain shift, and the anatomic distortion after tumor resection. This means the surgeon can then navigate to the area of residual tumor quickly and efficiently. This is particularly helpful in tumors where there is no overt tumor plane, or where the appearances are similar to that of normal tissue (for example low grade glioma).

IoMRI can also identify hemorrhage not immediately visible in the operative field; since the introduction of ioMRI, there have been no returns to theater for post-operative hemorrhage in our unit.

Nevertheless, ioMRI also has limitations: setup and running cost is a major prohibitory factor. A two room setup (as in our unit) can offset this, as the scanner can be used as a routine diagnostic tool when not in use intraoperatively (11). Another ioMRI suite option involves a ceiling mounted, moveable ioMRI scanner between two operating theaters (17).

While there is limited evidence in support of ioMRI in the pediatric neuro-oncology practice, an RCT in adults receiving craniotomy for glioma resection has been performed: the ioMRI group had 96% complete resection vs. 68 in the non ioMRI group (*p* = 0.02) (18).

#### Endoscopy

Technological advances in neuro-endoscopy have improved illumination, image resolution, and field of view (19). Recent technological developments include the use of smartphone integrated endoscopes (20), and the exoscope (21). The role and applicability of these emerging techniques in pediatric neuro-oncology surgery will become clearer as technology becomes more widely available.

The range of neuro-endoscopes offer different qualities making each one more appropriate for certain procedures. In our center, rigid endoscopy is performed exclusively. However, flexible neuro-endoscopy has been described for use in tumor excision (22).

Endoscopic (or endoscopic assisted) surgery may be used during the following: intraventricular surgery [tumor biopsy (23) tumor resection (24), perioperative hydrocephalus management including endoscopic third ventriculostomy (25)], endoscopic endo-nasal-trans-sphenoidal surgery (for resection of sella lesions) (26, 27), supra-cerebellar infra-tentorial endoscopic approaches to pineal region tumors, (28) and as an adjunct to the microscope during open craniotomy.

**Figure 3:**
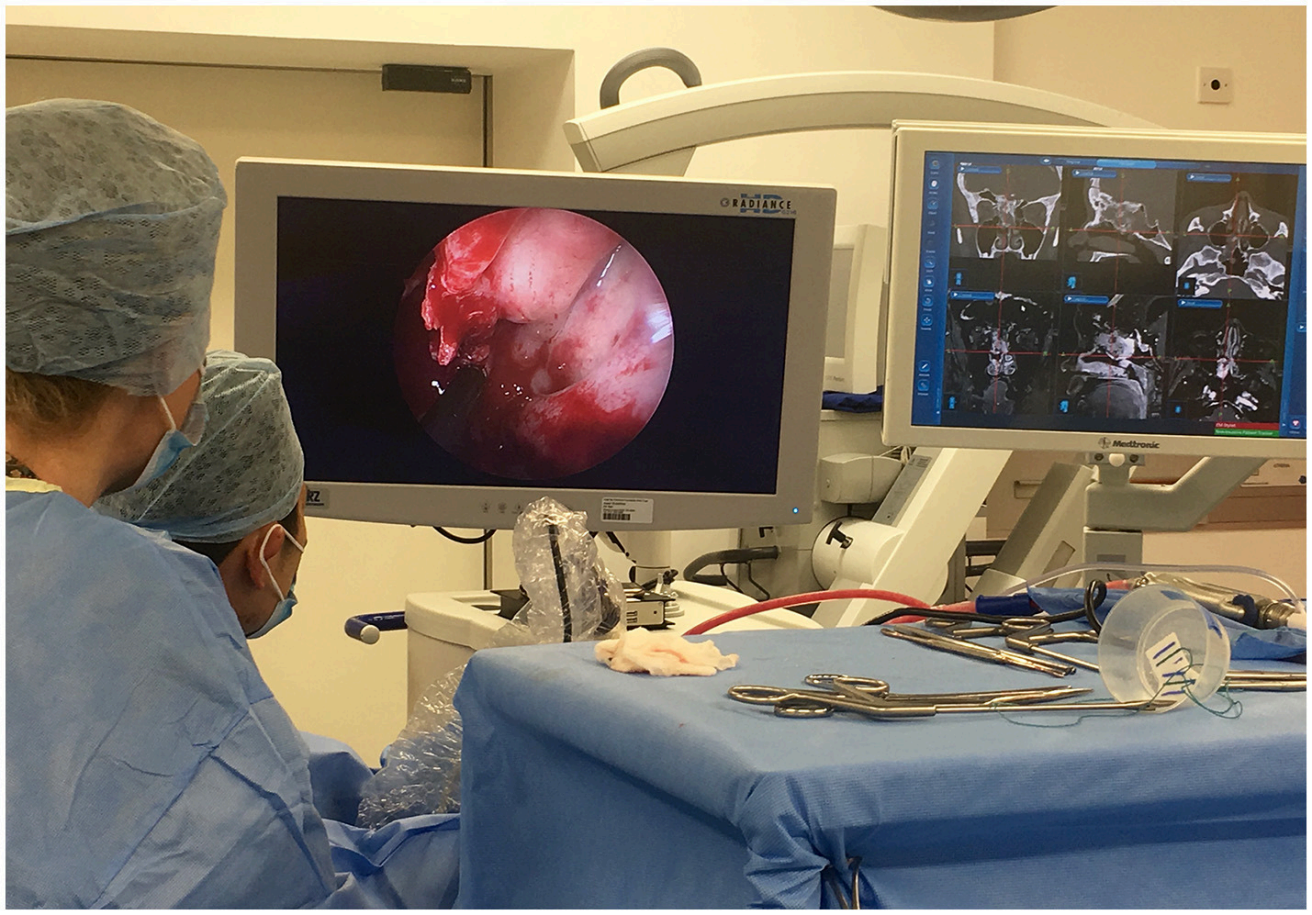
Multimodal use of technology: The use of intraoperative endoscopy for an extended endo-nasal approach, in tandem with neuro-navigation and intraoperative MRI was vital in maximizing resection of this complex recurrent atypical meningioma.

Pure endoscopic tumor resection is described for select cases, but is seldom performed in our practice, and is better described elsewhere (29, 30). The dexterity of endoscopic instruments and techniques is improving; the advent of endoscopic lasers, and ultrasonic aspirators has expanded the capability of endoscopic tumor resection (31). Endoscopy can be combined in a multimodal approach for example alongside neuronavigation and ioMRI (Figure 3).

#### Ultrasound

2D or 3D ultrasound can be used as a standalone tool (32), or in tandem with MRI for neuro-navigation (33). It allows real-time visualization of the parenchymal and ventricular anatomy. Doppler USS can be performed to assess vessels if required. US is also helpful in identifying normal ventricular anatomy which may be dynamic. It has limitations, being user dependent with a learning curve, susceptibility to artifact and non-uniform resolution (33). Echogenicity of tumors can vary depending on their type, meaning ioUS may sometimes be of little use. Furthermore, the walls of the resection cavity may be hyperechoic which can lead to overestimation of tumor residual (11).

#### Intraoperative fluorescence

Intra-operative fluorescence techniques can occasionally be used in pediatric neuro-oncology surgery. Orally administered 5-aminolevulinic acid (5-ALA) induces fluorescent porphyrin accumulation within certain tumors, which can be visualized with a modified microscope (34). The use of 5-ALA in pediatric patients is off label, but has been described (11, 35). An RCT in adults found 5-ALA improved the extent of tumor resection and benefitted progression free survival (36), however evidence in pediatric surgery is limited. Roth et al. noted that fluorescence is only seen in a small proportion of pediatric brain tumors (outside of glioblastoma multiforme), and therefore advise against the routine use of 5-ALA (35).

Indocyanine green is used in vascular neurosurgery to provide fluorescence to vessels directly visible in the operative microscope (11). This can be useful in pediatric neuro-oncology surgery if the tumor is especially vascular, or in close proximity to vital vascular structures (11).

The use of other fluorescence agents has also been described, including fluorescein, hypericin, 5-aminofluorescein-human serum albumin, and endogenous fluorophores, albeit with less evidence, especially in pediatrics (37).

Intraoperative Raman spectroscopy is an emerging technique with promise in distinguishing normal from pathological tissue (38). It has recently been described *in vivo* for core needle “biopsy” and delineation of pathological vs. normal tissue within resection cavity margins in a pig model (39).

#### Ultrasonic aspirator

There are various ultrasonic aspirators available that utilize ultrasound to emulsify and aspirate tissues (11). The weak intracellular bonds and high liquid content of tumor tissue make it susceptible to ultrasonic aspiration. Conversely, vessels and nerves with higher elastin and collagen content are less likely to be damaged (11).

#### Intraoperative neuromonitoring (ION)

Neuromonitoring intraoperatively is considered to be the gold standard in localizing brain function in brain tumor surgery (40). Mapping is the process of identifying the proximity to eloquent areas in the brain and avoiding damage to these regions (41). Both cortical and subcortical mapping can be performed (42). In pediatric neurosurgery where most of the supratentorial tumors are low grade lesions, the use of neuromonitoring helps in maximizing the extent of safe resection with least possible morbidity. Continuous, dynamic subcortical mapping with a suction monopolar device has recently been described (43).

Neurophysiological monitoring can also be used during resection of intramedullary spinal tumors by acquisition of data to confirm the integrity of neural pathways in the form of Motor evoked potentials (MEPs), D-waves and Somatosensory evoked potentials (SSEPs) (41, 44).

When using these techniques, total intravenous anesthesia is preferred, with avoidance of neuromuscular paralysis (11). Intraoperative neurophysiology is different in children to adults (especially infants), and requires and age adjustment to the stimulation techniques and interpretation of results because of the immaturity of the developing brain (41).

Awake craniotomy can be performed to allow real-time monitoring of neurological deficit alongside with cortical and subcortical mapping (45). However, this is seldom performed in children, although it has been described (46, 47).

Though there is sufficient evidence to support the use of ION in *predicting* neurological injury, there isn't much evidence for injury *prevention* (40).

## Improved understanding of neuroscience and neuroanatomy

Neuroanatomy education has benefitted from the capability of both 3D digital, and 3D printed models (48). In general, the interest in, and sophistication of general neurosurgical simulation is continually increasing (49), however there remains a need for more sophisticated tools to accurately replicate the challenges of pediatric neuro-oncology surgery.

Recent deeper understanding of the mechanisms behind cerebellar mutism following posterior fossa surgery have led to refinements in surgical technique to avoid damage to the proximal efferent cerebellar pathway (50).

## Discussion

Surgery has progressed through advances in the following areas: visualization (microscopes, endoscopes), Navigation (ioUS, MRI), and delineation of tumor resection (ioMRI, ioUS, 5ALA), all of which, in tandem can improve the ability to carry out maximal safe resection. However, many more major advances in pediatric neuro-oncology are non-surgical: the molecular classification of tumors, and the advances in chemotherapy and radiotherapy. The impact of these on surgical decision making is complex, and a full discussion of is beyond the scope of this article. However, one notable paradigm shift is seen in with molecular subcategorization of Medulloblastoma: gross total resection conferred no survival advantage in comparison with near total resection when taking into account molecular subgroups (51). Therefore, re-look surgery for residual disease is now not necessarily recommended if the risk of neurological morbidity from complete excision is high (51, 52). In contrast, the survival benefit from complete resection of Ependymoma is established (53, 54), and current consensus opinion is that molecular subcategorization should not change this surgical decision making (52, 55) As a result, re-look surgery for residual disease is recommended, and may involve referral to a quaternary center such as ourselves for second opinion.

In our unit, we use a combination of the following imaging modalities to delineate tumor resection: pre-operative MRI, intraoperative macroscopic, and microscopic assessment of tissues, intraoperative ultrasound, and intraoperative MRI. Since the introduction of the ioMRI in our unit, there have been no returns to theater for post-operative haematoma following craniotomy for tumor.

The best methodology for ensuring maximal safe tumor resection continues to be debated; however it is clear that no operative tool is flawless in isolation. MRI (preoperative or intraoperative) has superior anatomical resolution to ultrasound, but is a static, historic image. Conversely, ultrasound is in real-time, but is very user dependant, with variable tumor echogenicity. Advanced pediatric neuro-oncology surgery therefore utilizes a multimodal approach with navigation, microscopy, endoscopy if appropriate, and ioUS. The combination of tools with varying strengths can offset the limitations of tools also in use.

While the use of state of the art equipment discussed here has advanced pediatric neuro-oncology surgery, the importance of the perioperative and intraoperative MDT cannot be understated: without a complete, competent team (neuro-anesthetists, theater staff, nursing staff, neuro-radiologists, physiotherapy, occupational therapy, speech and language therapy, oncologists, endocrinologists, neurologists), outcomes would be compromised.

## Author contributions

MF drafted article. LH critically appraised and edited. CM is a lead surgeon in unit, critically appraised and edited.

### Conflict of interest statement

The authors declare that the research was conducted in the absence of any commercial or financial relationships that could be construed as a potential conflict of interest. The reviewer AD and handling Editor declared their shared affiliation.

## References

[1] Colafati GS Voicu IP Carducci C Miele E Carai A Di Loreto S. MRI features as a helpful tool to predict the molecular subgroups of medulloblastoma: state of the art. Ther Adv Neurol Disord. (2018) 11:1–14. 10.1177/1756286418775375 29977341 PMC6024494

[2] Perreault S Ramaswamy V Achrol AS Chao K Liu TT Shih D. MRI surrogates for molecular subgroups of medulloblastoma. Am J Neuroradiol. (2014) 35:1263–69. 10.3174/ajnr.A3990 24831600 PMC4819007

[3] Marcus HJ Vakharia VN Ourselin S Duncan J Tisdall M Aquilina K Robot-assisted stereotactic brain biopsy: systematic review and bibliometric analysis. Childs Nerv Syst. (2018) 34:1299–1309. 10.1007/s00381-018-3821-y PMC5996011 29744625

[4] Verploegh IS Volovici V Haitsma IK Schouten JW Dirven CM Kros JM. Contemporary frameless intracranial biopsy techniques: Might variation in safety and efficacy be expected? Acta Neurochir. (2015) 157:2011–16; discussion 2016. 10.1007/s00701-015-2543-0 26315461 PMC4604498

[5] Gempt J Buchmann N Ryang YM Krieg S Kreutzer J Meyer B. Frameless image-guided stereotaxy with real-time visual feedback for brain biopsy. Acta Neurochir. (2012) 154:1663–7. 10.1007/s00701-012-1425-y 22847726

[6] Silva MA See AP Essayed WI Golby AJ Tie Y. Challenges and techniques for presurgical brain mapping with functional MRI. Neuroimage Clin. (2017) 17:794–803. 10.1016/j.nicl.2017.12.008 29270359 PMC5735325

[7] Altman NR Bernal B. Pediatric applications of functional magnetic resonance imaging. Pediatr Radiol. (2015) 45(Suppl. 3):S382–96. 10.1007/s00247-015-3365-1 26346144

[8] Ekstrand CL Mickleborough MJ Fourney DR Gould LA Lorentz EJ Ellchuk T. Pre-surgical integration of fMRI and DTI of the sensorimotor system in transcortical resection of a high-grade insular astrocytoma. Front Integr Neurosci. (2016) 10:15. 10.3389/fnint.2016.00015 27013996 PMC4786563

[9] Farquharson S Tournier JD Calamante F Fabinyi G Schneider-Kolsky M Jackson GD. White matter fiber tractography: why we need to move beyond DTI. J Neurosurg. (2013) 118:1367–77. 10.3171/2013.2.JNS121294 23540269

[10] Mahvash M Boettcher I Petridis AK Besharati Tabrizi L. Image guided surgery versus conventional brain tumor and craniotomy localization. J Neurosurg Sci. (2017) 61:8–13. 10.23736/S0390-5616.16.03142-8 25600554

[11] Zebian B Vergani F Lavrador JP Mukherjee S Kitchen WJ Stagno V. Recent technological advances in pediatric brain tumor surgery. CNS Oncol. (2017) 6:71–82. 10.2217/cns-2016-0022 28001090 PMC6027926

[12] Zeiss Zeiss Kinevo 900 (2018). Available online at: https://www.zeiss.com/meditec/int/products/neurosurgery/surgical-microscopes/kinevo-900.html (Accessed July 24, 2018).

[13] Barone DG Lawrie TA Hart MG Image guided surgery for the resection of brain tumours. Cochrane Database Syst Rev. (2014) 28:CD009685 10.1002/14651858.CD009685.pub2 PMC6457761 24474579

[14] Jenkinson MD Barone DG Bryant A Vale L Bulbeck H Lawrie TA. Intraoperative imaging technology to maximise extent of resection for glioma. Cochrane Database Syst Rev. (2018) 1:CD012788. 10.1002/14651858.CD012788.pub2 29355914 PMC6491323

[15] Meola Cutolo F Carbone M Cagnazzo F Ferrari M Ferrari V. Augmented reality in neurosurgery: a systematic review. Neurosurg Rev. (2017) 40:537–48. 10.1007/s10143-016-0732-9 27154018 PMC6155988

[16] Tejada S Avula S Pettorini B Henningan D Abernethy L Mallucci C. The impact of intraoperative magnetic resonance in routine pediatric neurosurgical practice-a 6-year appraisal. Childs Nerv Syst. (2018) 34:617–26. 10.1007/s00381-018-3751-8 29460065

[17] Chicoine MR Lim CC Evans JA Singla A Zipfel GJ Rich KM. Implementation and preliminary clinical experience with the use of ceiling mounted mobile high field intraoperative magnetic resonance imaging between two operating rooms. Acta Neurochir Suppl. (2011) 109:97–102. 10.1007/978-3-211-99651-5_15 20960327

[18] Senft C Bink A Franz K Vatter H Gasser T Seifert V. Intraoperative MRI guidance and extent of resection in glioma surgery: a randomised, controlled trial. Lancet Oncol. (2011) 12:997–1003. 10.1016/S1470-2045(11)70196-6 21868284

[19] Li KW Nelson C Suk I Jallo GI. Neuroendoscopy: past, present, and future. Neurosurg Focus (2005) 19:E1. 10.3171/foc.2005.19.5.4 16398474

[20] Mandel M Petito CE Tutihashi R Paiva W Abramovicz Mandel S Gomes Pinto FC Smartphone-assisted minimally invasive neurosurgery. J Neurosurg. (2018) 13:1–9. 10.3171/2017.6.JNS1712 29529913

[21] Sack J Steinberg JA Rennert RC Hatefi D Pannell JS Levy M. initial experience using a high-definition 3-dimensional exoscope system for microneurosurgery. Oper Neurosurg Hagerstown Md (2018) 14:395–401. 10.1093/ons/opx145 29106670

[22] Ishikawa T Takeuchi K Tsukamoto N Kawabata T Wakabayashi T. A novel dissection method using a flexible neuroendoscope for resection of tumors around the aqueduct of sylvius. World Neurosurg. (2018) 110:391–6. 10.1016/j.wneu.2017.11.044 29158099

[23] Depreitere B Dasi N Rutka J Dirks P Drake J. Endoscopic biopsy for intraventricular tumors in children. J Neurosurg. (2007) 106(Suppl.):340–6. 10.3171/ped.2007.106.5.340 17566198

[24] Ahmad F Sandberg DI. Endoscopic management of intraventricular brain tumors in pediatric patients: a review of indications, techniques, and outcomes. J Child Neurol. (2010) 25:359–67. 10.1177/0883073809340318 20189934

[25] Fritsch MJ Doerner L Kienke S Mehdorn HM. Hydrocephalus in children with posterior fossa tumors: role of endoscopic third ventriculostomy. J Neurosurg. (2005) 103(1 Suppl.):40–2. 10.3171/ped.2005.103.1.0040 16122003

[26] Zhan R Xin T Li X Li W Li X. Endonasal endoscopic transsphenoidal approach to lesions of the sellar region in pediatric patients. J Craniofac Surg. (2015) 26:1818–22. 10.1097/SCS.0000000000001946 26352366 PMC4568898

[27] Alalade AF Ogando-Rivas E Boatey J Souweidane MM Anand VK Greenfield JP. Suprasellar and recurrent pediatric craniopharyngiomas: expanding indications for the extended endoscopic transsphenoidal approach. J Neurosurg Pediatr. (2018) 21:72–80. 10.3171/2017.7.PEDS17295 29125446

[28] Snyder R Felbaum DR Jean WC Anaizi A. Supracerebellar infratentorial endoscopic and endoscopic-assisted approaches to pineal lesions: technical report and review of the literature. Cureus 9:e1329. 6. 10.7759/cureus.1329 28690962 PMC5501715

[29] Barber SM Rangel-Castilla L Baskin D. Neuroendoscopic resection of intraventricular tumors: a systematic outcomes analysis. Minim Invasive Surg. (2013) 2013:898753. 10.1155/2013/898753 24191196 PMC3804403

[30] Rocque BG. Neuroendoscopy for intraventricular tumor resection. World Neurosurg. (2016) 90:619–20. 10.1016/j.wneu.2015.12.010 26721614 PMC5460758

[31] Cinalli G Imperato A Mirone G Di Martino G Nicosia G Ruggiero C. Initial experience with endoscopic ultrasonic aspirator in purely neuroendoscopic removal of intraventricular tumors. J Neurosurg Pediatr. (2017) 19:325–32. 10.3171/2016.10.PEDS16352 28084922

[32] Moiyadi V Shetty P. Direct navigated 3D ultrasound for resection of brain tumors: a useful tool for intraoperative image guidance. Neurosurg Focus 40:E5 (2016). 10.3171/2015.12.FOCUS15529 26926063

[33] Sastry R Bi WL Pieper S Frisken S Kapur T Wells W. Applications of ultrasound in the resection of brain tumors. J Neuroimaging (2017) 27:5–15. 10.1111/jon.12382 27541694 PMC5226862

[34] Stummer W Novotny A Stepp H Goetz C Bise K Reulen HJ. Fluorescence-guided resection of glioblastoma multiforme by using 5-aminolevulinic acid-induced porphyrins: a prospective study in 52 consecutive patients. J Neurosurg. (2000) 93:1003–13. 10.3171/jns.2000.93.6.1003 11117842

[35] Roth J Constantini S 5ALA in pediatric brain tumors is not routinely beneficial. Childs Nerv Syst. (2017) 33:787–92. 10.1007/s00381-017-3371-8 28293736

[36] Stummer W Pichlmeier U Meinel T Wiestler OD Zanella F Reulen HJ. Fluorescence-guided surgery with 5-aminolevulinic acid for resection of malignant glioma: a randomised controlled multicentre phase III trial. Lancet Oncol. (2006) 7:392–401. 10.1016/S1470-2045(06)70665-9 16648043

[37] Senders JT Muskens IS Schnoor R Karhade AV Cote DJ Smith TR. Agents for fluorescence-guided glioma surgery: a systematic review of preclinical and clinical results. Acta Neurochir. (2017) 159:151–67. 10.1007/s00701-016-3028-5 27878374 PMC5177668

[38] Broadbent B Tseng J Kast R Noh T Brusatori M Kalkanis SN. Shining light on neurosurgery diagnostics using Raman spectroscopy. J Neurooncol. (2016) 130:1–9. 10.1007/s11060-016-2223-9 27522510 PMC9308128

[39] Desroches J Jermyn M Pinto M Picot F Tremblay MA Obaid S. A new method using Raman spectroscopy for *in vivo* targeted brain cancer tissue biopsy. Sci Rep. (2018) 8:1792. 10.1038/s41598-018-20233-3 29379121 PMC5788981

[40] Coppola Tramontano V Basaldella F Arcaro C Squintani G Sala F. Intra-operative neurophysiological mapping and monitoring during brain tumour surgery in children: an update. Childs Nerv Syst. (2016) 32:1849–59. 10.1007/s00381-016-3180-5 27659828

[41] Kim K Cho C Bang M Shin H Phi JH Kim SK. Intraoperative neurophysiological monitoring : a review of techniques used for brain tumor surgery in children. J Korean Neurosurg Soc. (2018) 61:363–75. 10.3340/jkns.2018.0078 29742889 PMC5957318

[42] Bello L Riva M Fava E Ferpozzi V Castellano A Raneri F. Tailoring neurophysiological strategies with clinical context enhances resection and safety and expands indications in gliomas involving motor pathways. Neuro Oncol. (2014) 16:1110–28. 10.1093/neuonc/not327 24500420 PMC4096171

[43] Moiyadi Velayutham P Doctor J Borkar A Singh V. Continuous dynamic subcortical mapping using a suction monopolar device in a child: case report and technical note. J Pediatr Neurosci. (2018) 13:279–82. 10.4103/JPN.JPN_148_17 30090158 PMC6057198

[44] Chatterjee S Chatterjee U. Intramedullary tumors in children. J Pediatr Neurosci. (2011) 6(Suppl.1):S86–90. 10.4103/1817-1745.85718 22069435 PMC3208914

[45] Duffau H Peggy Gatignol ST Mandonnet E Capelle L Taillandier L. Intraoperative subcortical stimulation mapping of language pathways in a consecutive series of 115 patients with Grade II glioma in the left dominant hemisphere. J Neurosurg. (2008) 109:461–71. 10.3171/JNS/2008/109/9/0461 18759577

[46] Akay Rükşen M Çetin HY Seval HÖ Işlekel S. Pediatric awake craniotomy for brain lesions. Pediatr Neurosurg. (2016) 51:103–8. 10.1159/000442988 26783744

[47] Balogun JA Khan OH Taylor M Dirks P Der T Carter Snead O III. Pediatric awake craniotomy and intra-operative stimulation mapping. J Clin Neurosci. (2014) 21:1891–4. 10.1016/j.jocn.2014.07.013 25282393

[48] Arantes M Arantes J Ferreira MA. Tools and resources for neuroanatomy education: a systematic review. BMC Med Educ. (2018) 18:6. 10.1186/s12909-018-1210-6 29724217 PMC5934868

[49] Zhang L Kamaly I Luthra P Whitfield P. Simulation in neurosurgical training: a blueprint and national approach to implementation for initial years trainees. Br J Neurosurg. (2016) 30:577–81. 10.1080/02688697.2016.1211252 27601027

[50] Avula S Spiteri M Kumar R Lewis E Harave S Windridge D. Post-operative pediatric cerebellar mutism syndrome and its association with hypertrophic olivary degeneration. Quant Imaging Med Surg. (2016) 6:535–44. 10.21037/qims.2016.10.11 27942473 PMC5130563

[51] Thompson EM Hielscher T Bouffet E Remke M Luu B Gururangan S. Prognostic value of medulloblastoma extent of resection after accounting for molecular subgroup: a retrospective integrated clinical and molecular analysis. Lancet Oncol. (2016) 17:484–95. 10.1016/S1470-2045(15)00581-1 26976201 PMC4907853

[52] Guerreiro Stucklin S Ramaswamy V Daniels C Taylor MD. Review of molecular classification and treatment implications of pediatric brain tumors. Curr Opin Pediatr. (2018) 30:3–9. 10.1097/MOP.0000000000000562 29315108

[53] Merchant TE Li C Xiong X Kun LE Boop FA Sanford RA A prospective study of conformal radiation therapy for pediatric ependymoma. Lancet Oncol. (2009) 10:258–66. 10.1016/S1470-2045(08)70342-5 19274783 PMC3615425

[54] Sanford RA Merchant TE Zwienenberg-Lee M Kun LE Boop FA. Advances in surgical techniques for resection of childhood cerebellopontine angle ependymomas are key to survival. Childs Nerv Syst. (2009) 25:1229–40. 10.1007/s00381-009-0886-7 19484252

[55] Pajtler KW Mack SC Ramaswamy V Smith CA Witt H Smith A. The current consensus on the clinical management of intracranial ependymoma and its distinct molecular variants. Acta Neuropathol. (2017) 133:5–12. 10.1007/s00401-016-1643-0 27858204 PMC5209402

